# Editorial: Emerging treatment approaches for substance use disorders

**DOI:** 10.3389/fpsyt.2026.1926719

**Published:** 2026-07-21

**Authors:** David Martinez Garza, Ilya O. Blokhin, Jonathan Brett, Ihsan Salloum

**Affiliations:** 1 Department of Psychiatry and Behavioral Sciences, University of Miami, Miami, FL, United States; 2 Department of Psychiatry, Massachusetts General Hospital, Harvard University, Boston, MA, United States; 3 Department of Neuroscience, The University of Texas Rio Grande Valley, Edinburgh, TX, United States; 4 University of New South Wales, Kensington, NSW, Australia

**Keywords:** addiction, alcohol use disorder, emerging treatments, opioid use disorder, precision medicine, psychedelics, psychopharmacology, substance use disorders

Since the introduction of disulfiram in 1951 (the first ever Food and Drug Administration (FDA)-approved medication for substance use disorders (SUDs)) (1), only seven other individual medications have been approved for the treatment of SUDs. While the repurpose of medications and different formulations increase the total number of FDA-approved medicines [**Table 1** and (2)], the arsenal of pharmacological approaches remains limited for a group of diseases that affect 16.8% of individuals aged 12 or older (3), are linked to more than 700,000 deaths annually (4–6), and cost more than 700 billion dollars yearly (7). Notable progress in public health has been achieved with certain current medications [such as buprenorphine for opioid use disorder (8) or varenicline for nicotine use disorder (9)]. In contrast, attempts to identify successful, off-label treatment for other SUDs [topiramate for cocaine use disorder (10), bupropion with naltrexone for methamphetamine use disorder (11), or gabapentin for cannabis use disorder (12)] have yielded only relatively modest results.

**Table 1:** FDA-approved pharmacotherapy for SUDs (2).

Indication	Drug name	Year of approval
Alcohol Use Disorder	Disulfiram	1951
	Naltrexone	1994
	Acamprosate	2004
	Naltrexone (Injectable)	2006
Opioid Use Disorder	Methadone	1972
	Naltrexone	1984
	Buprenorphine	2002
	Naltrexone (Injectable)	2010
	Buprenorphine (Injectable) (a)	2017
Nicotine Use Disorder	Nicotine Replacement Therapy (b)	1984
	Bupropion	1997
	Varenicline	2006

a – Sublocade®(2017), Brixadi® (2023).

b – Gum (1984), Patch (1991), Nasal Spray (1996), Inhaler (1997), Lozenge (2002).

The current Research Topic is an effort to highlight emerging pharmacological and psychosocial treatments for SUDs. In recent years, several landmark papers have discussed the potential use of psychedelics in the treatment of SUDs [e.g. psilocybin for alcohol use disorder (13) or ketamine for cocaine use disorder (14)]. In this Research Topic, a study by Qeadan et al. analyzed a national cohort of patients with SUDs and found that psychedelic treatment (primarily ketamine) significantly improved outcomes, including relapse, overdose, and hospitalization, especially when combined with outpatient services, supporting the need for research on further integrated treatment. Shen et al. conducted a scoping review, which suggests that ketamine could be considered as a therapeutic agent in the management of opioid withdrawal as well as in reducing cravings in patients with opioid use disorder. In a case-study, Brett et al. described a patient who underwent psilocybin-assisted therapy for methamphetamine use disorder, with favorable outcomes on abstinence and overall mental health. Finally, in a large cross-sectional study, Glynos et al. found that more than two-thirds of participants with SUDs reported improved outcomes following the naturalistic use of psychedelics. These studies highlight the importance of future research evaluating the feasibility and efficacy of psychedelics in the treatment of SUDs. Outside of the realm of psychedelics, Alshehri, in a systematic review, proposes that valproic acid can reduce alcohol use, stabilize mood, and manage withdrawal in patients with co-occurring alcohol use disorder and psychiatric conditions.

Non-pharmacological interventions have also been employed to address SUDs, including cognitive behavioral therapy (CBT) (15), community reinforcement approach (16), motivational interviewing (17), and contingency management (18), with varying degrees of efficacy. In 2017, the FDA approved reSET^®^, a first-of-its-kind, prescription-only, 12-week digital course of CBT delivered directly to the patient’s smartphone for the treatment of SUDs (19). In 2018, reSET-O^®^, specifically designed for patients with opioid use disorder, was approved (20). In 2020, the FDA cleared Transcranial Magnetic Stimulation for short-term smoking cessation (21). In this Research Topic, Seok et al. conducted a meta-analysis on the effects of eye movement desensitization and reprocessing (EMDR) therapy on addiction-related symptoms, reporting the reduction of short-term cravings as well as a decrease in co-occurring symptoms such as depression and anxiety. Ssentongo et al. discussed how combining inpatient education and counseling with pharmacotherapy reduced readmission risk for patients with SUDs, advocating for the integration of addiction consultation services in hospitals.

To conclude, a thought-provoking series of articles explores emerging approaches to treating SUDs. Nikolova et al. introduce the Cognitive Dysfunction in the Addictions (CDiA) program, an interdisciplinary research initiative evaluating the impact of executive functions on functional outcomes in adults with SUDs. This ambitious project aims to identify personalized, targeted addiction treatments. Zafar et al. argue in favor of a focus on a biomarker-guided theragnostic framework when approaching SUDs, and review approaches aimed at the integration of neuroimaging into clinical trials of psychedelics to inform personalized therapeutic interventions. Finally, in a compelling perspective article, Unterrainer, using Icarus as a metaphor, examined addiction as a distorted, often self-destructive, pursuit of transcendence to fill an existential or spiritual void.

Taken together, this Research Topic highlights important areas of research in the field of treatment for SUDs, and accepted manuscripts suggest promising pharmacological and psychotherapeutic strategies and provide new foundational approaches to the disease as a whole. We thank the authors for submitting their interesting work to our Research Topic. We also express gratitude to our peer reviewers for the thoughtful and rigorous evaluations. Lastly, but no less importantly, we thank the editorial and production staff at *Frontiers in Psychiatry* for their support and meticulous management of the publication workflow and peer review process. We hope this Research Topic will spark interest and encourage further studies that will expand our knowledge on emerging therapeutic options for patients with SUDs.
